# Inhibition of Bruton’s tyrosine kinase interferes with pathogenic B-cell development in inflammatory CNS demyelinating disease

**DOI:** 10.1007/s00401-020-02204-z

**Published:** 2020-08-06

**Authors:** Sebastian Torke, Roxanne Pretzsch, Darius Häusler, Philipp Haselmayer, Roland Grenningloh, Ursula Boschert, Wolfgang Brück, Martin S. Weber

**Affiliations:** 1 Institute of Neuropathology, University Medical Center, Göttingen, Germany; 2 Department of Neurology, University Medical Center, Göttingen, Germany; 3 Translational Innovation Platform Immunology, Merck KGaA, Darmstadt, Germany; 4 EMD Serono Research and Development Institute, Inc., Billerica, MA USA; 5 Ares Trading S.A., An Affiliate of Merck Serono S.A., Eysins, Switzerland

**Keywords:** Multiple sclerosis, Experimental autoimmune encephalomyelitis, Bruton’s tyrosine kinase, BTKi, Evobrutinib, B cells, Myeloid cells

## Abstract

Anti-CD20-mediated B-cell depletion effectively reduces acute multiple sclerosis (MS) flares. Recent data shows that antibody-mediated extinction of B cells as a lasting immune suppression, harbors the risk of developing humoral deficiencies over time. Accordingly, more selective, durable and reversible B-cell-directed MS therapies are needed. We here tested inhibition of Bruton’s tyrosine kinase (BTK), an enzyme centrally involved in B-cell receptor signaling, as the most promising approach in this direction. Using mouse models of MS, we determined that evobrutinib, the first BTK inhibiting molecule being developed, dose-dependently inhibited antigen-triggered activation and maturation of B cells as well as their release of pro-inflammatory cytokines. Most importantly, evobrutinib treatment functionally impaired the capacity of B cells to act as antigen-presenting cells for the development of encephalitogenic T cells, resulting in a significantly reduced disease severity in mice. In contrast to anti-CD20, BTK inhibition silenced this key property of B cells in MS without impairing their frequency or functional integrity. In conjunction with a recent phase II trial reporting that evobrutinib is safe and effective in MS, our mechanistic data highlight therapeutic BTK inhibition as a landmark towards selectively interfering with MS-driving B-cell properties.

**Electronic supplementary material:**

The online version of this article (10.1007/s00401-020-02204-z) contains supplementary material, which is available to authorized users.

## Introduction

Over the last decade, the perception of the role that B cells play in the pathogenesis and propagation of multiple sclerosis (MS) fundamentally changed [14, 19, 25]. While earlier concepts focused on the ability of B cells to differentiate into antibody-secreting plasma cells providing the diagnostic hallmark of intrathecally produced oligoclonal bands [27], B cells themselves are now considered to be important pathogenic drivers of the disease [25]. Recent evidence suggests that one key property of B cells in MS is their ability to specifically recognize antigen via their B-cell receptor (BCR), and to present the internalized, processed antigen via MHC class II to responding T cells [15]. This concept was substantiated by the clinical success of anti-CD20 antibodies in clinical MS trials, in which depletion of peripheral B cells promptly halted development of new central nervous system (CNS) MS lesions [2, 12, 13, 16, 23]. Notwithstanding this fundamental achievement, the extinction of a relatively small fraction of highly differentiated, antigen-specific B cells is relevant to clinical stabilization [1], while the larger population of CD20 positive B cells, including subsets with regulatory properties desirable to be retained in MS [5, 14], are collaterally deleted [18]. Furthermore, since MS is a chronic disease requiring long-term treatment, continuous pan-B-cell depletion with an increasing risk of humoral immunosuppression over time may not be a durable strategy to control pathogenic B-cell function over decades [3, 7, 21, 22, 24].

Based on these limitations of anti-CD20 therapy, together with an improved mechanistic understanding how B-cell subsets trigger MS activity, novel B-cell-directed MS treatments are being pioneered. One promising approach is the inhibition of Bruton’s tyrosine kinase [20], an enzyme centrally involved in B-cell activation upon BCR antigen recognition [8]. Besides B-cell signaling, BTK is also crucial for Fc receptor (FcR) mediated activation of myeloid cells [4, 8], an alternative pathway to facilitate antigen recognition via antibody-mediated opsonization. Based on this mechanism of action not relying on the depletion of immune cells, various BTK inhibitors are being developed for treatment of MS. The first-in-class of these agents is evobrutinib, a highly selective, covalent BTK inhibitor [11], which has generated promising results in a recent phase II MS trial [20]. In the study reported here, we aimed to provide the mechanistic counterpart to this pivotal clinical trial by dissecting the effects of evobrutinib in a mouse model of MS, and by translating the derived observations back to MS patients’ immune cells.

## Methods

### Approval of human sampling

Human peripheral blood mononuclear cells (PBMCs) were obtained after informed consent. The protocol was approved by the Ethics committee of the University Medicine of Göttingen, approval number 3/4/14.

### Mice

Wild type (WT) C57BL/6 mice were bred in-house. MOG p35-55 TCR transgenic 2D2 mice were kindly provided by Dr. Kuchroo (Boston, USA). All animal experiments were carried out in accordance with the guidelines of the Central Department for Animal Experiments, University Medical Center, Göttingen, and approved by the Office for Consumer Protection and Food Safety of the State of Lower Saxony (protocol number 33.9-42502-04-16/2267).

### Evobrutinib dosing and exposure

Evobrutinib was formulated in 20% Kleptose HPB in 50 mM Na-Citrate buffer pH 3.0 and administered by oral gavage. One hour after dosing, blood was collected via the vena facialis into serum tubes. The tubes were centrifuged, serum was collected and used for measurement of evobrutinib concentration by LC–MS.

### EAE induction and scoring

Female WT mice were immunized subcutaneously with 75 µg MOG protein 1–117 emulsified in Complete Freund’s Adjuvant containing 250 µg killed *Mycobacterium tuberculosis* H37 Ra followed by intraperitoneal injections of 300 ng of *Bordetella pertussis* toxin on the day of immunization and 2 days thereafter. EAE severity was assessed daily on a scale from 0 to 5 (0 = no clinical signs; 1.0 = tail paralysis; 2.0 = loss of righting reflex; 3.0 = beginning hind limb paresis; 4.0 = paralysis of both hind limbs; 4.5 = beginning forelimb paresis 5.0 = moribund/death).

### Histology and immunohistochemistry

Mice were perfused transcardially with PBS followed by 4% paraformaldehyde and tissue was paraffin embedded. Sections (1 μm) were stained with hematoxylin and eosin (HE) and Luxol fast blue/periodic acid shift. T cells, B cells and macrophages were detected by immunohistochemistry with an avidin–biotin technique using antibodies specific for CD3 (clone SP7), CD45R/B220 (clone RA3-6B2) and Mac-3 (clone M3/84). Histological sections were captured using a digital camera mounted on a light microscope or a VS120 slide scanner. The percentage of demyelinated white matter was calculated using ImageJ. Overall immune cell infiltration was assessed on HE stained slides using an automated counting macro. Inflammatory cells were quantified at 400 × magnification using an ocular counting grid and are shown as cells/mm^2^. At least 4 spinal cord cross sections were taken for each analysis.

### Isolation of human and murine leucocytes

PBMCs from healthy donors were isolated after Ficoll gradient centrifugation. Human B cells were purified from PBMCs by positive MACS separation using a human CD19 isolation kit or negative separation using the memory B-cell isolation kit or B-cell isolation kit II. Single cell suspensions of murine lymphoid tissues were generated by gentle dissection and passing through a 70 µm cell strainer. Murine blood was collected in PBS containing 1 mM EDTA followed by erythrocyte lysis using BD Pharm Lysing Buffer. Murine B and T cells were isolated by negative MACS separation using a mouse pan T-cell isolation kit II or positive MACS separation using a MojoSort mouse B-cell isolation.

### Flow cytometry

Murine immune cells was analyzed using the following antibodies: CD3 (clone 145-2C11), CD4 (clone GK1.5), CD8 (clone 53-6.7), CD11b (clone M1/70), CD19 (clone 6D5; clone 1D3), CD21 (clone 7G6), CD23 (clone B3B4), CD25 (clone PC61.5), CD27 (clone LG.3A10), CD44 (clone IM7), CD45R/B220 (clone RA3-6B2), CD69 (clone H1.2F3), CD80 (clone GL1), CD86 (clone GL-1), CD93 (clone AA4.1), IgD (clone 11-26c.2a), IgM (clone AF6-78) and MHCII (clone AF6-120.1).

Human immune cells were analyzed using the following antibodies: BTK (clone 53/BTK), pBTK (clone N35-88), CD19 (clone HIB19), CD27 (clone L128), CD38 (clone HIT-2), IgD (clone IA6-2), IgM (clone MHM-88).

For the analysis of T-cell proliferation, cells were stained with carboxyfluorescein succinimidyl ester (CFSE). T regulatory cell differentiation was evaluated by intracellular staining for FoxP3 (clone FJK-16s) after fixation and permeabilization using the fixation/permeabilization kit. To investigate Th1 and Th17 cell differentiation cells were stimulated with 50 ng/ml phorbol 12-myristate 13-acetate and 0.5 µg/ml ionomycin for 3 h with subsequent addition of 1 µl/ml brefeldin A for 2 h. Cytokine production was analyzed by intracellular staining for IFN-γ (clone XMG1.2) and IL-17A (clone TC11-18H10) after fixation/permeabilization. Dead cells were stained with LIVE/DEAD™ Fixable Aqua Dead Cell Stain Kit. Samples were acquired on a BD LSR Fortessa. All data evaluation was performed using FlowJo software.

### Calcium flux

Purified B or T cells were stained in complete HBSS medium (HBSS medium containing 1.3 mM CaCl_2_, 0.5 mM MgCl_2_ and 1% FCS) containing 0.02% Pluronic F-68 at 37 °C for 30 min in the presence of 4 mg/ml Fluo-3 AM and 10 mg/ml Fura Red AM. Cells were kept on ice, pre-incubated with the indicated concentrations of evobrutinib for at least 30 min, and directly before flow cytometry acquisition pre-heated to 37 °C for 5 min. After 25 s baseline recording, human or murine B cells were stimulated with 10–40 µg/ml human or 5–20 µg/ml murine anti-IgM/anti-IgG F(ab’)_2_ fragment (Dianova). Murine T cells were marked using antibodies against CD3 (clone 145-2C11) and CD28 (clone 37.51) which, after baseline recording, were crosslinked using 5 µg/ml anti-hamster secondary antibody. Human T cells were stimulated using 5 µg/ml ionomycin.

### Quantitative PCR

Isolated B cells were incubated with evobrutinib for 60 min at 37 °C followed by stimulation with 10 µg/ml anti-IgM/IgG F(ab’)_2_ fragment at 37 °C for 3 h. RNA was isolated using the RNeasy mini kit and transcribed into cDNA using the QuantiNova Reverse Transcription kit. Quantitative (q)PCR was performed using 500 nM Primer and qPCRBIO SyGreen in a total volume of 10 µl on a QuantStudio 7. Primers specific for IL-6, IL-10, IFNγ, B2M and GAPDH were purchased from Eurofins Genomics. qPCR was performed at 95 °C denaturating and 70 °C annealing temperature for 30 s and 40 cycles with subsequent melt-curve analysis. Primer specificity was validated by product size using a 2% Agarose gel containing GelRed and UV-light illumination. Detailed primer information is listed in the Supplementary Table 2, online resource. Samples were analyzed in duplicate or triplicate and considered valid when cycle threshold (*C*_t_) < 35 and standard deviation of *C*_t_ < 0.5. Analyzed cytokine expression was normalized to B2M and GAPDH (delta-*C*_t_). The relative expression was determined in comparison to the control-treated group.

### Stimulation human B cells

Isolated human B cells were left to rest for 1 h and pre-incubated with the indicated concentrations of evobrutinib for at least 30 min before stimulation with 10 µg/ml anti-IgM F(ab’)_2_ fragment or 4 µg/ml CpG for 22 h in duplicates. Supernatants were collected after centrifugation and stored at − 20 °C before detection of IL-6, interferon-γ and IL-10 by ELISA according to the manufacturers’ instructions. Cytokine production was normalized to vehicle control.

### Statistical analysis

Statistics were calculated using the software GraphPad Prism 6.01. The clinical score, maturation, qPCR and human flow cytometric data were analyzed by 2-way ANOVA. Histology, calcium flux, T-cell proliferation, T-cell differentiation, cytokine production were analyzed by ordinary 1-way ANOVA. Expression of activation markers was analyzed by Kruskal–Wallis. All groups were compared to control treatment and corrected for multiple comparisons.

## Results

### Evobrutinib ameliorates clinical and histological experimental autoimmune encephalomyelitis (EAE)

We first investigated the effectiveness of evobrutinib in a B-cell-accentuated version of the murine experimental autoimmune encephalomyelitis (EAE) MS model. In the setting used, B cells recognize the conformationally folded immunogen myelin oligodendrocyte glycoprotein (MOG) [26]. BCR ligation results in cellular activation as well as internalization and subsequent presentation of the antigen to T cells [26]. Accordingly, this model is considered to reflect the pathogenic contribution of antigen-activated B cells. Mice were treated once daily with 1, 3, or 10 mg/kg evobrutinib; a dose of 3 mg/kg was previously shown to completely block disease development in mouse models of rheumatoid arthritis (RA) and systemic lupus erythematosus (SLE), and to achieve a near-complete BTK inhibition [11].

**Fig. 1:**
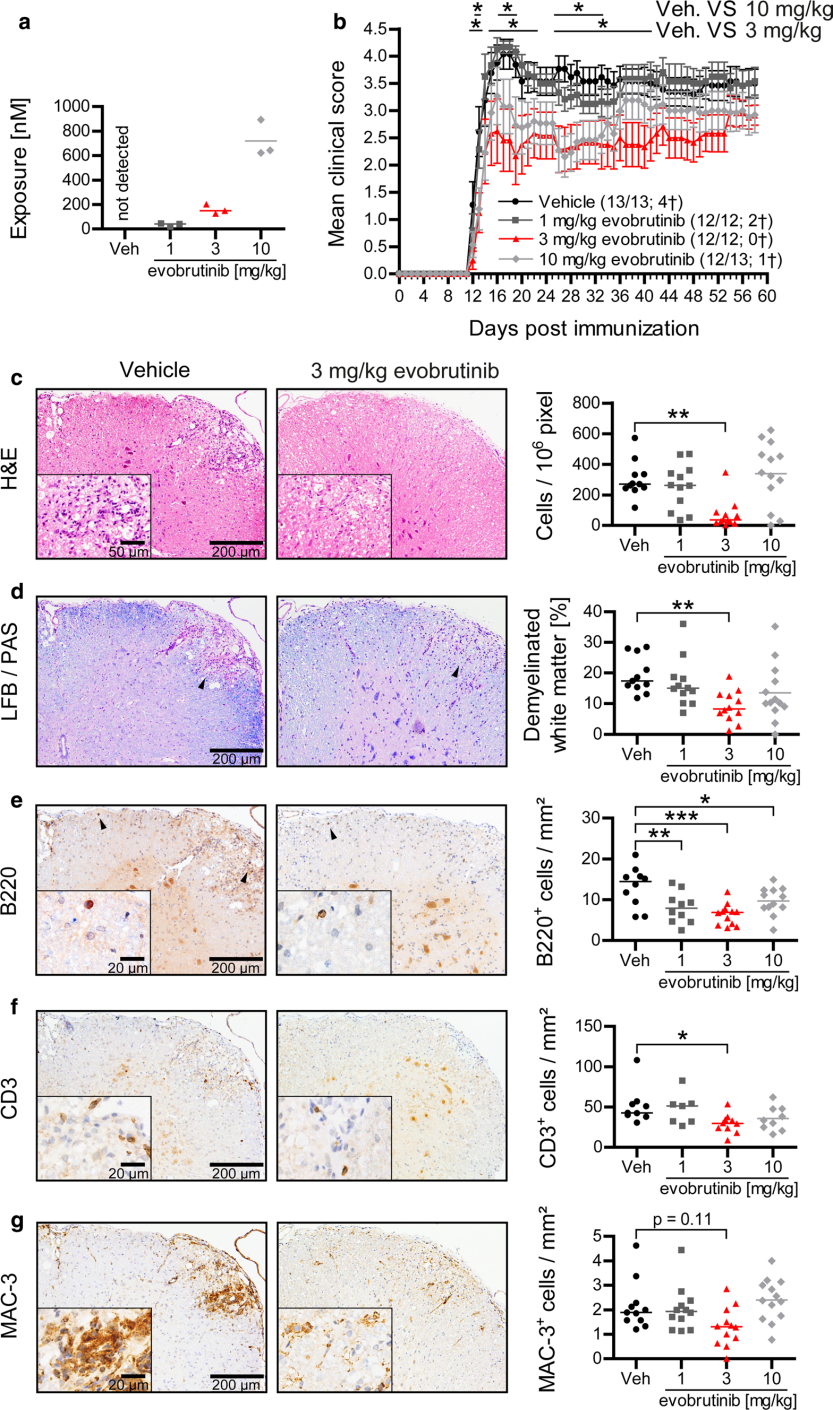
Evobrutinib reduces clinical and histological EAE. Oral treatment of C57/Bl6 mice with vehicle or 1, 3 or 10 mg/kg evobrutinib started 7 days prior to immunization with 75 µg conformational MOG1-117 protein. **a** Exposure was measured in the serum 1 h after oral dosing by LC–MS. **b** Clinical severity was assessed on a standard 0–5 scale. Spinal cords were isolated 60 days after EAE induction and stained for **c** overall immune cell infiltration (HE), **d** overall demyelination (LFB/PAS) and the infiltration of **e** B cells (B220^+^), **f** T cells (CD3^+^) and **g** macrophages (Mac3). Mean ± SEM or median is shown, *n* = 9–13, *p < 0.05, **p < 0.01, ***p < 0.001

As shown in Fig. 1a, daily oral evobrutinib treatment resulted in very consistent, dose-dependent blood exposure levels reflecting a range from partial to complete BTK inhibition as determined in an earlier experimental study [11]. Using prophylactic treatment starting 7 days before immunization, low-dose evobrutinib showed no clinical and limited histological effects. Importantly, however, the intermediate dose (3 mg/kg) strongly ameliorated clinical and histological EAE, determined by overall immune cell infiltration into the CNS and by the extent of demyelination (Fig. 1b–d). Of note, the extent of clinical score benefit observed with evobrutinib was comparable to that of pan-B-cell depletion (Supplementary Fig. 1, online resource). No additional benefit was observed with increased dose, supporting the notion of complete inhibition at 3 mg/kg and suggesting that the observed efficacy was solely mediated by BTK inhibition. Furthermore, the mortality rate of 30% in control-treated mice was reduced to 0% in the evobrutinib intermediate-dose group (Fig. 1b). Immunohistochemistry revealed a pronounced decline of CNS B-cell infiltrates, a relative reduction of CNS T cells and an unchanged frequency of MAC-3 positive myeloid cells (Fig. 1c–g). Together, these observations suggested that evobrutinib controlled the severity of clinical and histological EAE by a direct effect on B cells and a possibly indirect effect on T cells.

### Evobrutinib blocks antigen-driven maturation of B cells

**Fig. 2:**
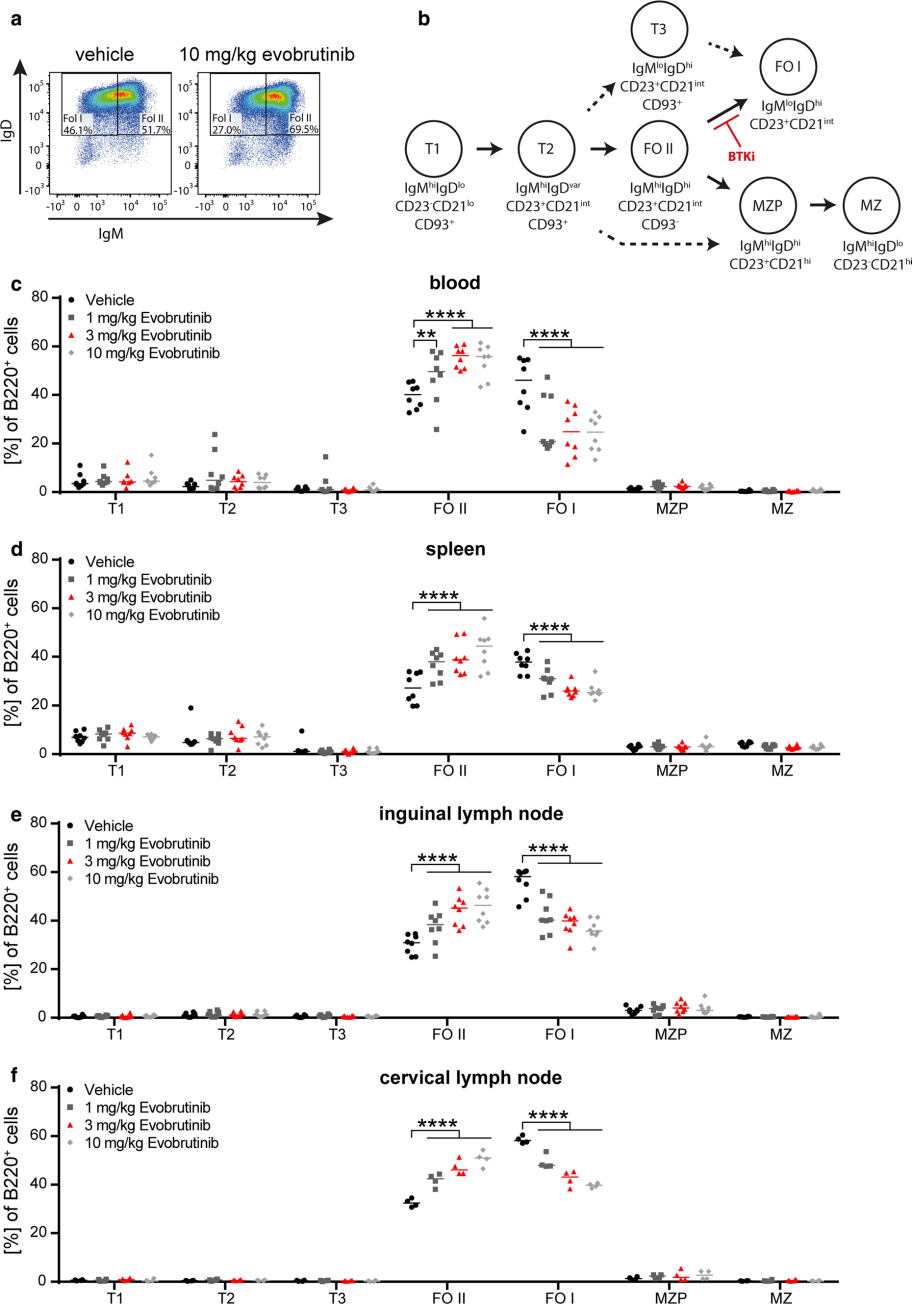
Evobrutinib inhibits the maturation of B cells. Oral treatment of C57/BL6 mice with vehicle or 1, 3 or 10 mg/kg evobrutinib started 7 days prior to immunization with 75 µg conformational MOG1-117 protein. Single cell suspensions of the secondary immune organs were analyzed 12 days after immunization by flow cytometry. **a**, **b** B-cell subsets were categorized into transitional (T), follicular (FO), marginal zone precursor (MZP) and marginal zone (MZ) cells. Frequency of B220^+^ cells is shown for **c** blood, **d** spleen, **e** inguinal lymph node and **f** cervical lymph node. Median, *n* = 8–16 pooled from 4 to 8 independent experiments, **p < 0.01, ****p < 0.0001

Based on these clinical and histological observations, we next investigated the phenotype, activation status and function of B cells in this setting. Mice were treated with evobrutinib and immunized as before. Importantly, we confirmed that BTK inhibition is not associated with a decline in the frequency of B cells in blood or any secondary lymphoid organs (Supplementary Fig. 2a, online resource). While we observed a relative increase in the frequency of T cells in the inguinal lymph node, the site of the immunization, the T-cell frequencies in blood, spleen and the cervical lymph node remained unaltered (Supplementary Fig. 2b, c, online resource). Of note, the absolute numbers of B cells and T cells in the spleen were not affected by evobrutinib treatment (Supplementary Fig. 2d, e, online resource). Next, we assessed maturation of B cells in the blood, lymph nodes and spleen by assessing the percentage of T1, T2, T3, follicular (FO) I/II B cells, marginal zone precursors or marginal zone B cells in each compartment (Supplementary Fig. 2f, online resource). Strikingly, we observed a dose-dependent accumulation of FO II B cells and a corresponding reduction of FO I B cells in mice treated with evobrutinib in all investigated organs, while none of the other subsets were changed in their frequency (Fig. 2). These data suggest that differentiation of B cells into an activated phenotype was broadly affected by evobrutinib in all immunologically relevant compartments.

### Evobrutinib inhibits expression of molecules involved in B-cell antigen presentation

**Fig. 3:**
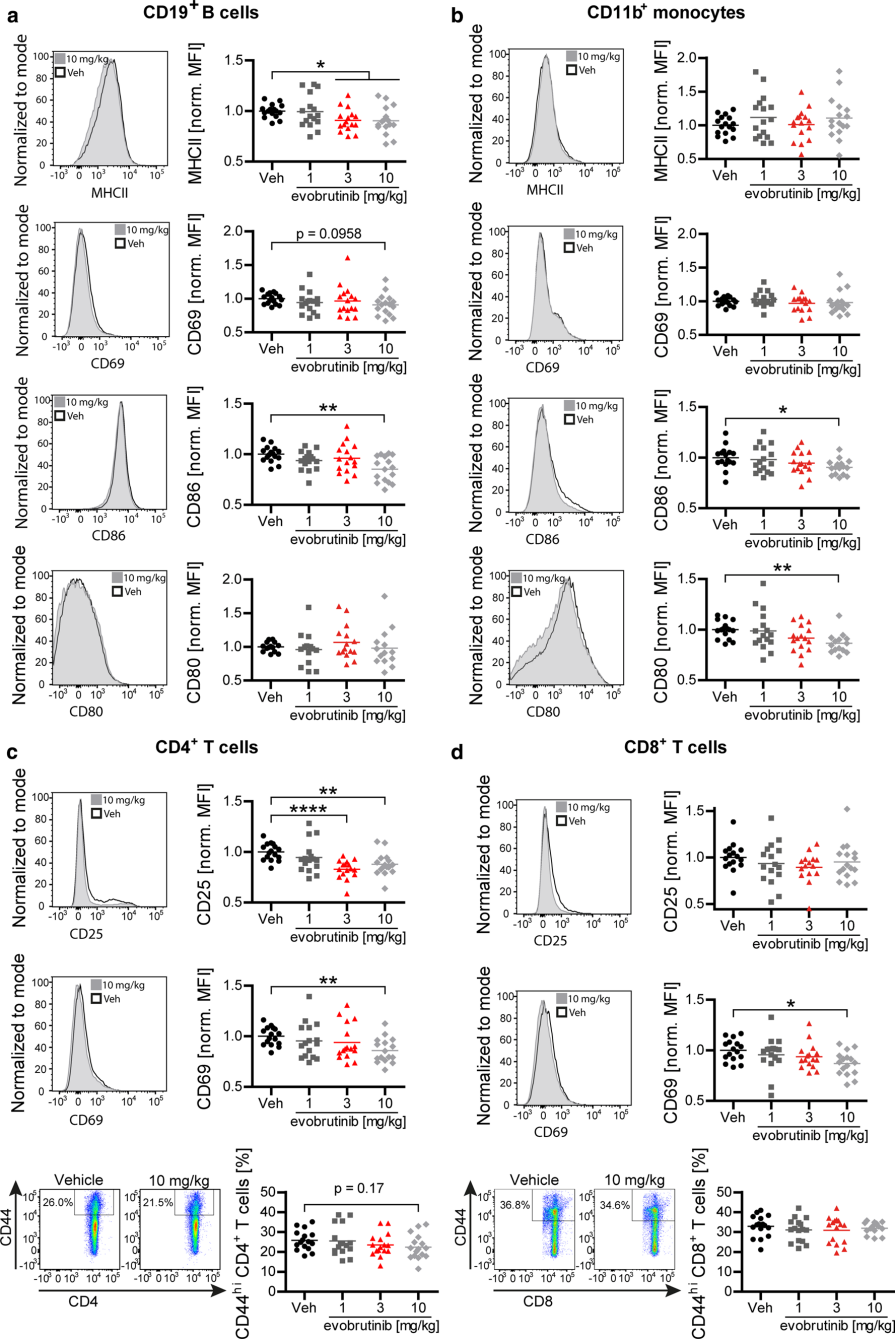
Evobrutinib reduces expression of activation markers on B and T cells. Oral treatment of C57/BL6 mice with vehicle or 1, 3 or 10 mg/kg evobrutinib started 7 days prior to immunization with 75 µg conformational MOG1-117 protein. Splenic cells were analyzed 12 days after immunization by flow cytometry. Expression of MHCII, CD69, CD86 and CD80 on **a** B cells and **b** Monocytes. Expression of CD25 or CD69 and frequency of CD44^hi^ cells of **c)** CD4^+^ T cells and **d** CD8^+^ T cells. Median, *n* = 16 pooled from 4 to 8 independent experiments, *p < 0.05, **p < 0.01, ****p < 0.0001

In parallel, the B-cell expression of MHC class II and co-stimulatory CD86, both essential for antigen presentation, were reduced by evobrutinib treatment, while the level of CD80 was unchanged (Fig. 3a). We next investigated CD11b^+^ myeloid cells, which serve as alternative antigen-presenting cells (APCs) in this model, and detected a reduction in the expression of CD80 and CD86, while levels of MHCII and CD69 were unchanged (Fig. 3b). CD4^+^ T cells, which are the main effector cells in EAE, showed substantially reduced activation as assessed by the expression of CD25 and CD69 and a trend towards a lower frequency of CD44^hi^ expressing memory T cells (Fig. 3c). CD8^+^ T cells showed only a reduced expression of CD69 (Fig. 3d). Since T cells do not express BTK, we attributed these effects on T cells to a reduced ability of evobrutinib-treated B cells to act as APCs and to promote the development of encephalitogenic T cells.

### Evobrutinib impairs the ability of B cells to generate encephalitogenic T cells

**Fig. 4:**
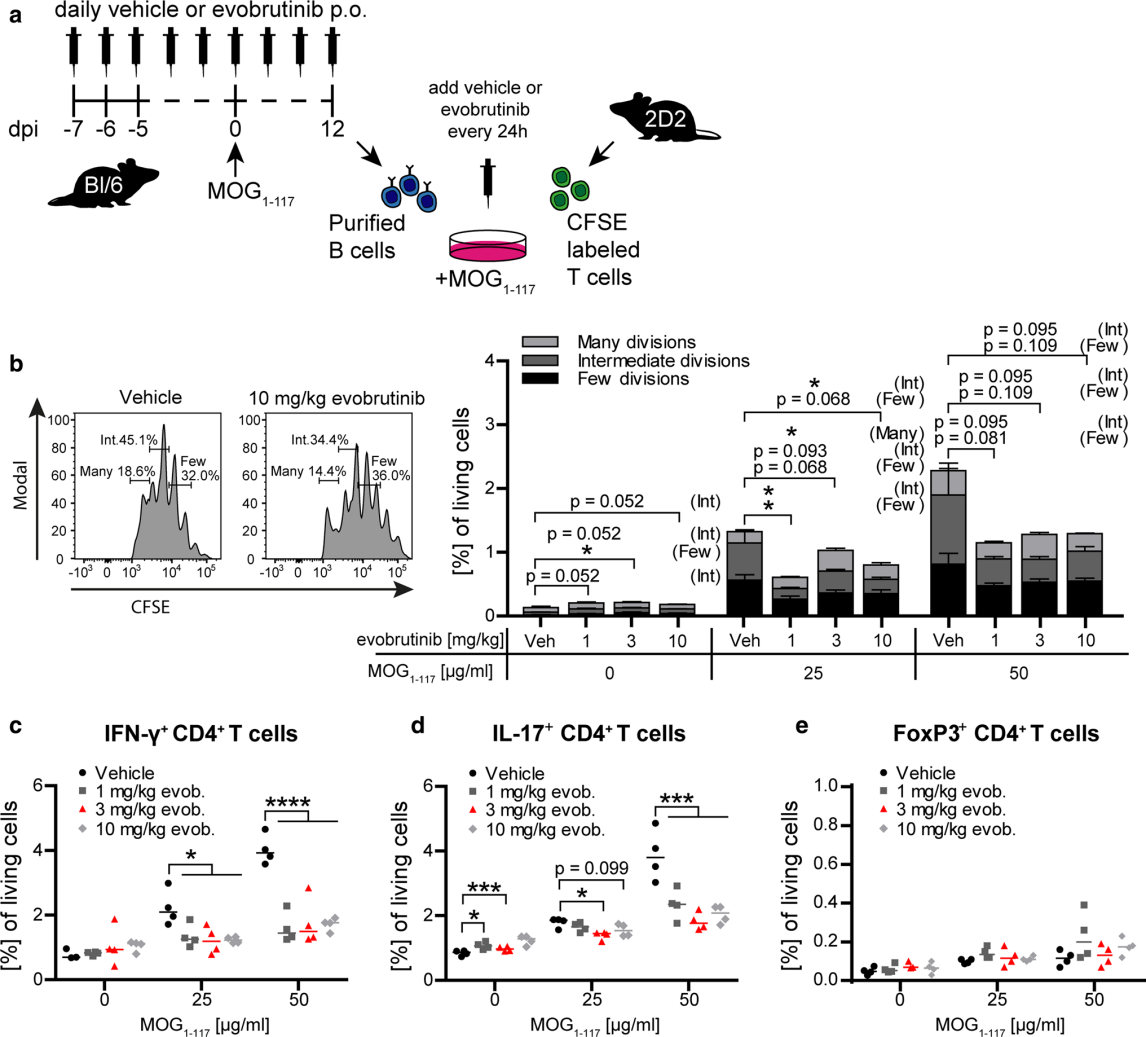
Evobrutinib inhibits B-cell APC function. Oral treatment of C57/BL6 mice with control or 1, 3 or 10 mg/kg evobrutinib started 7 days prior to immunization with 75 µg conformational MOG1-117 protein. **a** Splenic B cells were isolated from mice 12 days after immunization; T cells were obtained from unimmunized, untreated 2D2 mice. T cells were co-cultured with B cells for 72 h in the presence of the indicated concentrations MOG protein. Evobrutinib concentrations equivalent to serum levels 1 h after oral dosing were added every 24 h. **b** T cell proliferation was analyzed by CFSE dilution. T cell differentiation was analyzed by intracellular flow cytometry for the production of **c)** IFN-γ, **d** IL-17 and **e** FoxP3. Mean ± SEM or median, *n* = 4. Representative data from at least 2 independent experiments, *p < 0.05,***p < 0.001, ****p < 0.0001

To assess this hypothesis directly, we co-cultured B cells from evobrutinib-treated immunized animals with MOG-specific T cells isolated from naïve, non-evobrutinib-treated transgenic mice (Fig. 4a). Evobrutinib treatment of B-cell donor mice started 7 days prior to immunization and B cells were isolated 12 days later. First, we investigated to what extent BTK inhibition of B cells affected the proliferation of corresponding T cells. As indicated in Fig. 4b, overall T-cell proliferation was reduced when evobrutinib-treated B cells served as APCs. Most strikingly, differentiation of naïve T cells into pro-inflammatory Th1 and Th17 effector T cells was greatly diminished (Fig. 4c,  d), while the frequency of regulatory, FoxP3-expressing CD4^+^ T cells was unchanged (Fig. 4e). These findings confirm that evobrutinib-mediated BTK inhibition of B cells selectively affected their ability to generate encephalitogenic T cells.

### Evobrutinib directly interferes with BCR-mediated B-cell activation and differentiation

**Fig. 5:**
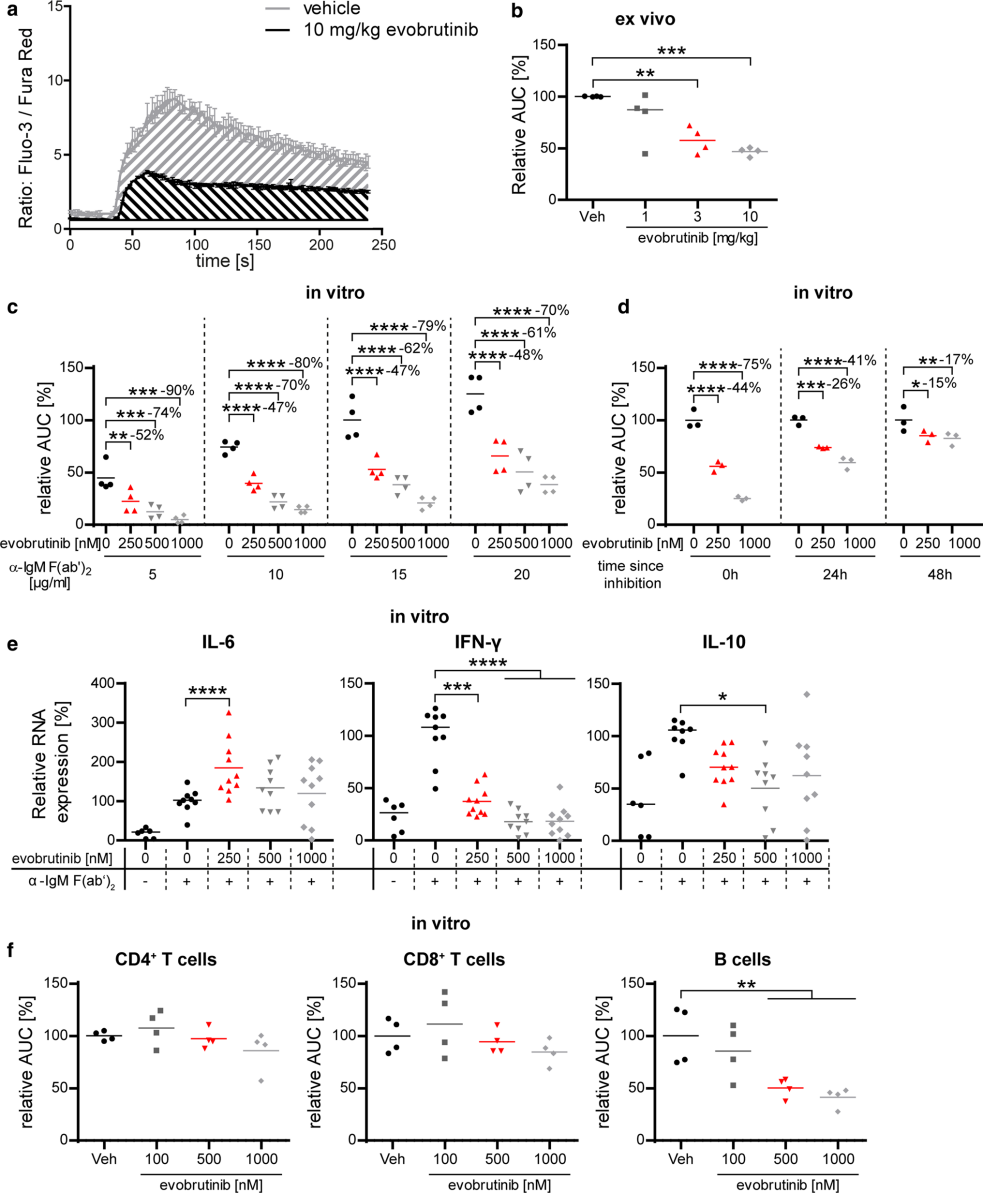
Evobrutinib specifically inhibits B cellular excitatory calcium mobilization and cytokine production. **a**, **b**. B cells were isolated from naïve C57/BL6 mice after 3 days of evobrutinib treatment and stained using Fluo-3 and Fura Red for calcium mobilization assay. After 25 s baseline recording, cells were stimulated with 20 µg/ml anti-IgM/anti-IgG. **c** Calcium mobilization on naïve murine B cells was analyzed after at least 30 min In vitro pre-incubation with the indicated concentrations evobrutinib. After 25 s baseline recording, cells were stimulated with the indicated concentrations anti-IgM/anti-IgG. **d** For BTKi reversibility, murine B cells were incubated for 30 min with the indicated concentrations evobrutinib and stained for calcium mobilization directly after as well as after 24 and 48 h. **e** Cytokine production was analyzed after 3 h anti-IgM/anti-IgG stimulation via qPCR. **f** B and T cells were pre-incubated with the indicated concentrations of evobrutinib and **s**timulated with anti-IgM/anti-IgG (B cells) or anti-CD3**/**anti-CD28 crosslinking (T cells). Median, *n* = 4–10, pooled from at least 2 independent experiments, *p < 0.05, **p < 0.01, ***p < 0.001, ****p < 0.0001

To better understand how BTK inhibition modulates B-cell function, we modelled BCR-mediated B-cell activation in the presence or absence of BTK inhibition by visualizing cellular mobilization of calcium upon BCR ligation (Fig. 5a). B cells were purified from naïve, healthy mice after 3 day treatment with vehicle or 1, 3 or 10 mg/kg evobrutinib (Fig. 5b); in a complementary in vitro setting, freshly isolated B cells from non-evobrutinib-treated mice were directly pre-incubated with evobrutinib doses up to 1 µM (Fig. 5c). Of note, the dose range used in this in vitro setting closely mirrored the serum levels measured in evobrutinib-treated mice (Fig. 1a). In both settings, we observed that evobrutinib strongly inhibited intracellular calcium mobilization in B cells upon BCR ligation via anti-IgM. Importantly, this inhibitory effect was strongly dose-dependent and could be partially overridden by increasing the strength of the stimulus. Of note, the inhibitory effect was reversible as functional BCR signaling was largely restored within 48 h, both in murine and human B cells (Fig. 5d, Supplementary Fig. 3, online resource). Next, we investigated, in the identical setting, to what extent BTK inhibition affected B-cell differentiation and production of cytokines. We detected relatively small changes in the levels of IL-6 and IL-10 mRNA. In contrast, the expression of IFN-γ mRNA was strongly upregulated upon BCR ligation, and this was dose-dependently reversed, and at higher doses completely blocked, by evobrutinib (Fig. 5e). To control for any toxic or potential off-target effect of evobrutinib, cells not expressing BTK were used as control. For this purpose, T cells were exposed to the identical doses of evobrutinib and then activated via anti-CD3/anti-CD28 stimulation in vitro. As shown in Fig. 5f, no inhibitory effect of evobrutinib on calcium mobilization was detected in either CD4^+^ or CD8^+^ T cells, supporting the conclusion that evobrutinib affected B-cell activation and cytokine production by a specific inhibitory effect on BTK.

### Evobrutinib inhibits activation of human B cells

**Fig. 6:**
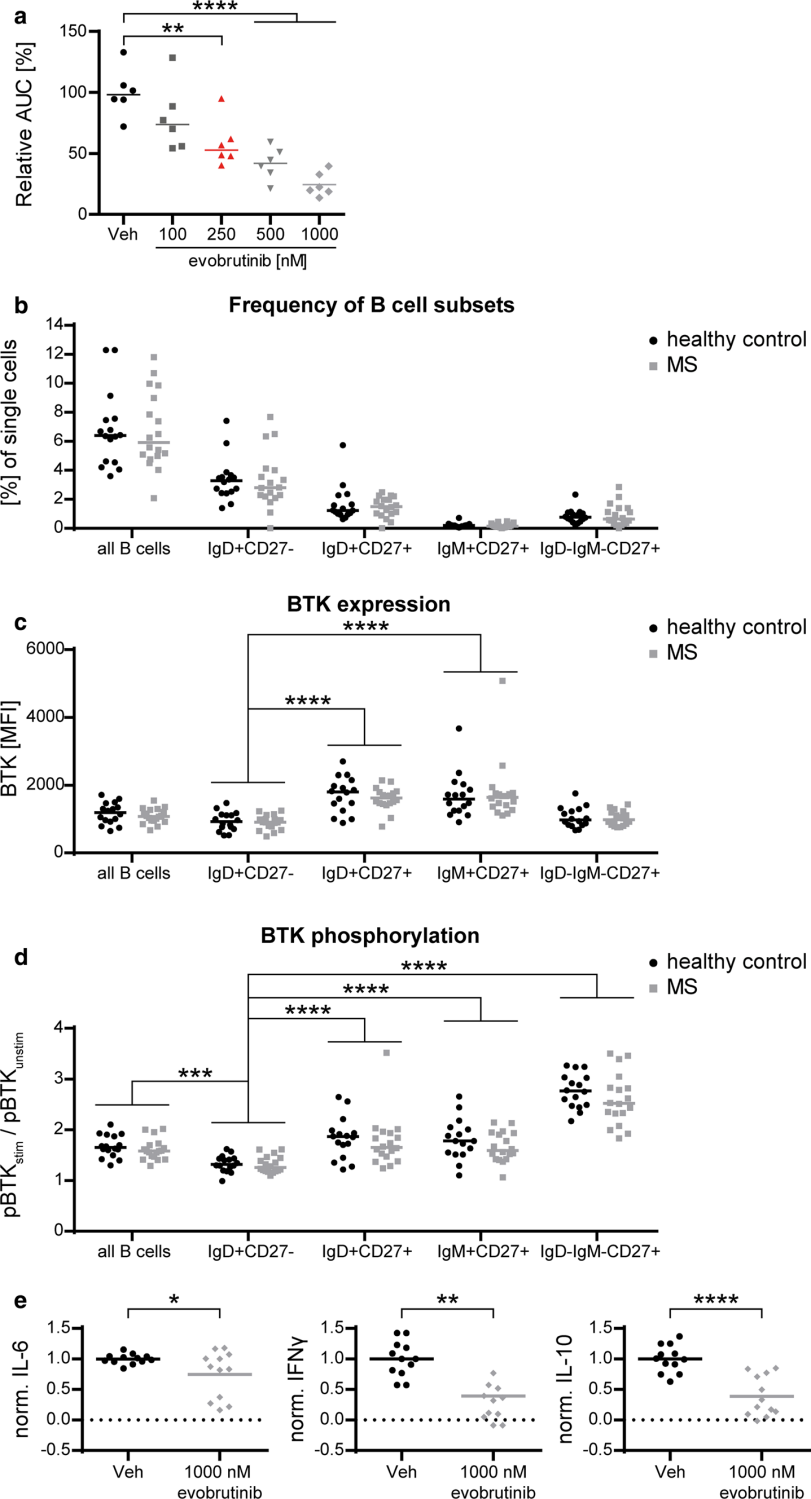
BTK inducibility depends on B-cell maturation and activation, evobrutinib reduces cytokine production. PBMCs from healthy controls (HC) or MS patients were isolated freshly for B-cell purification or thawed from − 80 °C storage. **a** Purified B cells were stained using Fluo-3 and Fura Red for calcium mobilization assay. After 25 s baseline recording, cells were stimulated with 40 µg/ml anti-IgM/anti-IgG. PBMCs stained for surface markers and stimulated for 30 s using anti-IgM/anti-IgG. After immediate fixation and permeabilization, intracellular antibodies for BTK and pBTK (Y223) were incubated for 1 h. **b** Frequency of B cells/B-cell subsets on single cells. **c** BTK expression by MFI **d** BTK phosphorylation inducibility. **e** Isolated B cells were stimulated using 10 µg/ml anti-IgM for 22 h. Cytokine production of IL-6, IFN-γ and IL-10 was analyzed by ELISA and normalized to vehicle control. Median, *n* = 6–18, pooled from at least 3 independent experiments, *p < 0.05, **p < 0.01, ***p < 0.001, ****p < 0.0001

We next assessed to what extent our mechanistic findings in mice mirrored the situation in humans. For this purpose, we exposed freshly isolated human B cells to the equivalent doses of evobrutinib. Strikingly, we observed that evobrutinib dose-dependently reduced calcium mobilization upon BCR ligation in a manner indistinguishable from the murine setting, while T cells again remained unaffected (Fig. 6a, Supplementary Fig. 4a, online resource). As a proof of principle, we compared the levels of inhibition in an MS patient and a healthy control, observing no variance in the overall calcium mobilization as well as the inhibitory effect of evobrutinib. (Supplementary Fig. 4b, online resource). This is an indication that there is no inherent difference between MS patients and healthy subjects in regard to calcium mobilization and/or BTK inhibition. However, since this is only one patient, these findings need to be substantiated by further experiments Next, we investigated the expression and activity of BTK in different B-cell subsets isolated from healthy individuals and MS patients (Supplementary Table 1, online resource). We categorized B cells into naïve (IgD^+^CD27^−^), activated but not class-switched (IgD^+^CD27^+^, IgM^+^CD27^+^) and fully class-switched (IgD^−^IgM^−^CD27^+^) subsets. Comparing healthy controls and patients with MS, we detected no difference in the frequency of B cells or B-cell subsets (Fig. 6b, Supplementary Fig. 4c, online resource). Furthermore, we observed no alteration in the relative expression of BTK between healthy and MS individuals. This is of interest by itself, as in other well characterized, B-cell-driven autoimmune diseases, such as anti-citrullinated protein antibody-positive RA, expression of BTK in B cells was increased [9]. However, we detected highly significant differences when various B-cell subsets were compared. CD27^+^ activated B cells generally expressed higher levels of BTK than the other subsets (Fig. 6c, Supplementary Figure 4d, online resource). Comparing the level of BTK activation (Tyr223 phosphorylation), B cells from MS patients and healthy controls were indistinguishable. However, we observed substantial differences in BTK phosphorylation between different B-cell subsets. Of note, phospho-BTK inducibility increased with B-cell differentiation and was relatively low in non-activated, naïve B cells (Fig. 6d) and absent in plasmablasts (Supplementary Fig. 4e, online resource). Next, we analyzed the effect of evobrutinib on cytokine production of activated human B cells. As shown in Fig. 6e, production of IL-6, IFN-γ and IL-10 upon BCR ligation was reduced by evobrutinib. A similar inhibitory effect was observed when B cells were stimulated alternatively via Toll-like receptor (TLR)-9 (Supplementary Figure 4f, g, online resource). Taken together, this human dataset indicates that BTK is preferentially expressed and functionally involved in more differentiated and class-switched B cells, indicating that evobrutinib primarily targets B-cell subsets with projected pathogenic potential in MS.

## Discussion

In this study, we investigated in detail the immunological effects of therapeutically blocking BTK with evobrutinib. In a mouse model actively involving B cells, this novel agent dose-dependently ameliorated clinical and histological EAE. Importantly, the inhibitory effect on clinical severity and histological parameters was strongest in the intermediate evobrutinib-treated group. There was an unexpected increase in clinical disease in the high-dose evobrutinib-treated animals which is still histologically detectable at the end of the study. Dissecting the clinical benefit, we found that BTK inhibition prevented transition of B cells from FO II to FO I phenotype, a key step in B-cell maturation which is known to heavily rely on BCR ligation and to be BTK dependent [28]. Evobrutinib treatment was further associated with reduced expression of MHC-II and co-stimulatory molecules. Of note, both the B-cell activation and maturation are dose-dependently inhibited, with the strongest inhibition in the highest treated animals. This apparent disparity between the immunological analysis and the clinical and histological EAE is one limitation of our study and remains to be further investigated. As a direct consequence of these B-cell-directed effects, the total population of B cells isolated from evobrutinib-treated mice was strongly diminished in their ability to activate naïve T cells and to promote development of encephalitogenic T cells when used as APCs. This indirect effect of evobrutinib on pathogenic T-cell development was similarly observed when T cells were directly isolated from evobrutinib-treated mice, indicating that preventing potent B-cell APC development may be a central mechanism underlying the clinical efficacy of evobrutinib in treatment of CNS demyelinating disease. In vivo evobrutinib treatment as well as in vitro exposure of purified B cells to evobrutinib dose-dependently inhibited BCR-mediated activation of B cells, which as a downstream effect reduced production of inflammatory cytokines. While we could demonstrate in principle that human B-cell function and activity was impaired in the same way, one limitation of our study is that we could not examine B-cell development, activation and differentiation in MS patients treated with evobrutinib, since clinical samples were not available. Nevertheless, in conjunction with the parallel experimental data and the observation that evobrutinib-mediated BTK inhibition broadly diminished antigen-triggered activation of human B cells, this overall “B-cell-silencing” effect mechanistically complements and underpins the observed clinical benefit of evobrutinib in MS [20].

These data are of fundamental interest as they help to better understand the effectiveness, but also to anticipate possible limitations, of evobrutinib in the treatment of MS, which, based on the positive phase II trial, [20] is now being advanced to Phase III evaluation. Although not formally tested head-to-head in our study, the extent of evobrutinib’s clinical benefit in B-cell-accentuated EAE was comparable to that seen with anti-CD20 treatment. Furthermore, the highlighted central mechanism of evobrutinib, involving inhibition of the ability of B cells to support the development of pathogenic T cells, is, in its downstream effect on T cells, similar to anti-CD20 treatment. However, an important difference between BTK inhibitors and antibody-based B-cell depletion is the comparatively smaller size of the inhibitors, which increases their potential to effectively penetrate the CNS [6]. Additionally, in contrast to anti-CD20, inhibition of BTK does not destroy or lastingly reduce the frequency of B cells [17]. Despite the fact that BTK inhibition affects all B-cell subsets which express BTK, the inhibition only becomes relevant upon specific receptor engagement, such as the BCR. Several immune system-controlling functions of B cells, however, are generated by resting, non-activated cells or plasmacells which do not rely on BCR signaling. Furthermore, the relative expression of BTK as well as its functional involvement in the BCR signaling cascade appears to increase with activation and class-switching. Taken together, these findings imply that BTK inhibition may exert a pronounced effect on differentiated B cells. While also not proven head-to-head, the anticipated effect size of evobrutinib in MS in the time-period observed so far, however, unlikely reaches the level of disease control achieved by anti-CD20-mediated depletion of B cells. This discrepancy may relate to known limitations of the EAE model and, in particular, to the timing of intervention. In our study, evobrutinib treatment was initiated prior to induction of EAE, to most suitably study its effect on pathogenic B-cell development. In MS, by contrast, disease-driving B-cell function is likely at least partly established at the time of initiating therapy. Accordingly, preventing de novo development of pathogenic B cells may not control the pathogenic properties of B cells as rapidly as pan-B-cell depletion, although the extent of disease control may increase over time. A somewhat opposite scenario applies to anti-CD20 therapy in which MS clinical activity is immediately silenced, while side effects, such as compromised humoral defense, may arise with its continuous use [3, 7, 21, 22, 24]. A possible approach to overcome the distinct potential limitations of each class of agents may be to use B-cell-eliminating anti-CD20 antibodies and B-cell-silencing BTK inhibitors in sequence [10]. Such a strategy would harness the immediate and thorough clinical effect of anti-CD20 while allowing the recurrence of B cells as the source of antibody-secreting plasma cells thereafter. Conversely, prior eradication of pathogenic B cells may enhance the effectiveness of evobrutinib, as preventing redevelopment of disease-driving B cells may then be as effective as continuous anti-CD20 treatment. Future clinical trials will have to assess the potential of this sequential combination of B-cell-directed MS agents.

In conclusion, our data establish and highlight the capability of therapeutic BTK inhibition to control disease-driving B-cell–T cell interactions in inflammatory CNS demyelination without permanently removing either cell type. This is demonstrated here for evobrutinib, the first BTK inhibitor clinically tested in MS, and the elucidated immunological effects may similarly apply to other BTK inhibitors in clinical development. Based on this advance, we believe that the mechanistic data provided here will be instrumental in guiding how this new class of innovative B-cell-directed agents are integrated into the current MS treatment landscape.

## Electronic supplementary material

Below is the link to the electronic supplementary material.

Supplementary material 1 (DOCX 34 kb)

Supplementary material 2 (TIFF 5716 kb)

Supplementary material 3 (TIFF 83165 kb)

Supplementary material 4 (TIFF 14576 kb)

Supplementary material 5 (TIFF 41591 kb)

## Data Availability

The data of this study are available upon reasonable request.
