# Supplementary material for: Inhibition of Bruton’s tyrosine kinase interferes with pathogenic B-cell development in inflammatory CNS demyelinating disease
Source: Acta Neuropathol. 2020 Aug 6;140(4):535–48. doi: 10.1007/s00401-020-02204-z (PMC7498502; doi:10.1007/s00401-020-02204-z)
Supplement: Supplementary file 1 — Supplementary material 1 (DOCX 34 kb) [file 401_2020_2204_MOESM1_ESM.docx]

**Supplementary material**

**Suppl. Fig. 1 Anti-CD20 mediated B cell depletion ameliorates EAE**

Prophylactic treatment of C57/Bl6 with control or anti-CD20 antibody started 3 weeks prior to immunization with 75 µg conformational MOG1-117 protein along with 300ng PTX on days 0 and 2. **a)** Clinical severity was assessed on a standard 0-5 scale. Mean±SEM, n=8-10

**Suppl. Fig. 2**

Oral treatment of C57/Bl6 with control or 1, 3 or 10 mg/kg evobrutinib started 7 days prior to immunization with 75 µg conformational MOG1-117 protein. Cells isolated from the various organs were analyzed 12 days after immunization by flow cytometry. **a)** Normalized frequency in the indicated organs and **b)** absolute cell counts in the spleen of B cells. **c)** Frequency and **d)** absolute cell counts for T cells in the spleen. **e)** B cell subsets were categorized into transitional (T), follicular (FO), marginal zone precursor (MZP) and marginal zone (MZ) cells. Median, n=4-8 pooled from 2-4 experiments.

**Suppl. Fig. 3**

B cells were isolated from C57/BL6 mice or healthy human subjects, incubated for 30 min with the indicated concentrations evobrutinib, washed and stimulated using 1 µg/ml CpG. The cells were then stained for calcium mobilization directly after as well as after 24 and 48 hours. After 25s baseline recording, cells were stimulated with10 µg/ml or 5 µg/ml anti-IgM/anti-IgG for murine and human cells, respectively. Median, n=2-3, one experiment, * p<0.05, ** p<0.01, **** p<0.0001.

**Suppl. Fig. 4**

PBMCs from healthy controls (HC) or MS patients were freshly purified or thawed from -80°C storage. **a+b)** Purified B or T cells were stained using Fluo-3 and Fura Red for calcium mobilization assay. After 25s baseline recording, cells were stimulated with 20 µg/ml anti-IgM/anti-IgG or 5 µg/ml ionomycin. **c-e)** Purified B cells were stained for surface markers and stimulated for 30s using anti-IgM. After immediate fixation and permeabilization, intra-cellular antibodies for BTK and pBTK (Y223) were incubated for 1h. **c)** Frequency of B cells / B cell subsets on single cells. **d)** BTK expression by MFI **e)** BTK phosphorylation inducibility. Isolated B cells were stimulated using 4 µg/ml CpG for 22h. Cytokine production of IL-6 (**f**) and IL-10 (**g**) was analyzed by ELISA. Median, n=10-18, pooled from at least 3 independent experiments, * p<0.05, ** p<0.01, *** p<0.001, **** p<0.0001.

**Suppl. Table 1 Patient characteristics**

|  | **HC** | **MS** | **p-value** |
| --- | --- | --- | --- |
| **Age Mean ± SD** | 37.75 ± 12.12 | 37.93 ± 13.39 | 0.9669 |
| **Female, n (%)** | 9 (56.25%) | 11 (61.11%) | 0.7738 |
| **Time since diagnosis [years],**  **mean ± SD** | n.a. | 4.81 ± 5.83 |  |
| **EDSS, mean ± SD** | n.a. | 3.35 ±2.37 |  |
| **Clinical course of MS, n (%)** |  |  |  |
| CIS / RIS | n.a. | 3 (16.67%) |  |
| RRMS | n.a. | 10 (55.55%) |  |
| SPMS / PPMS | n.a. | 5 (27.78%) |  |
| **Treatment** |  |  |  |
| Corticosteroids (within last 2m) | n.a. | 4 (22.22%) |  |
| DMD (>7d, within last 2m) | n.a. | 1 (5.55%, Copaxone) |  |
| untreated | n.a. | 14 (77.77%) |  |

**Suppl. Table 2 Primer Information**

| **Target** | **Amplicon length** | **Fw primer** | **Rv primer** |
| --- | --- | --- | --- |
| IL-6 | 152 | CCTCTGGTCTTCTGGAGTACC | ACTCCTTCTGTGACTCCAGC |
| IL-10 | 206 | ATAACTGCACCCACTTCCCA | GGGCATCACTTCTACCAGGT |
| IFN-gamma | 165 | TTCTTCAGCAACAGCAAGGC | TCAGCAGCGACTCCTTTTCC |
| B2M | 227 | CGGCCTGTATGCTATCCAGA | GGGTGAATTCAGTGTGAGCC |
| GAPDH | 83 | CATGGCCTTCCGTGTTCCTA | TGTCATCATACTTGGCAGGTTTCT |
