# Supplementary figures and images for: Inhibition of Bruton’s tyrosine kinase interferes with pathogenic B-cell development in inflammatory CNS demyelinating disease
Source: Acta Neuropathol. 2020 Aug 6;140(4):535–48. doi: 10.1007/s00401-020-02204-z (PMC7498502; doi:10.1007/s00401-020-02204-z)

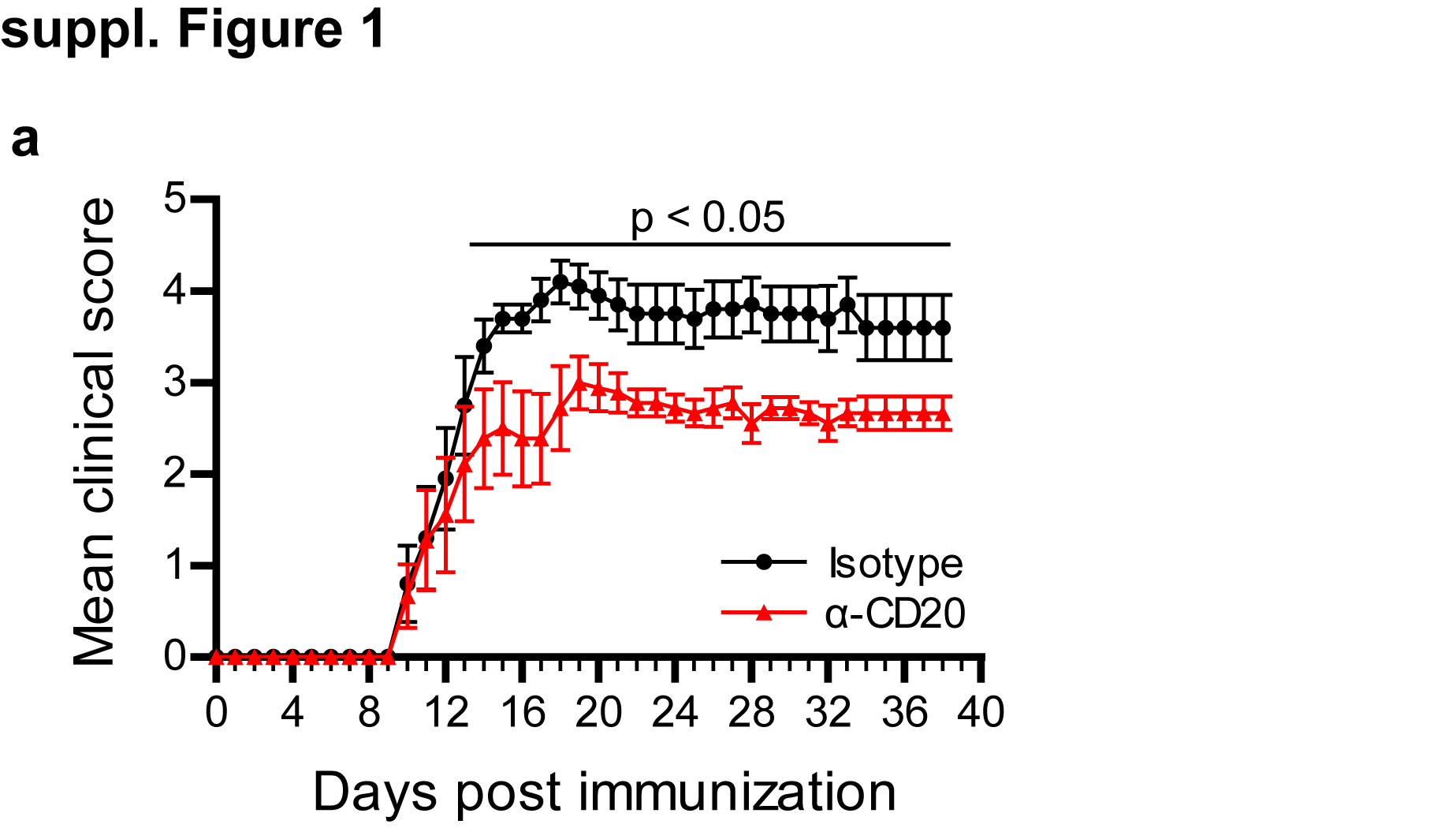

Supplement: Supplementary file 2 — Supplementary material 2 (TIFF 5716 kb) [file 401_2020_2204_MOESM2_ESM.tif]

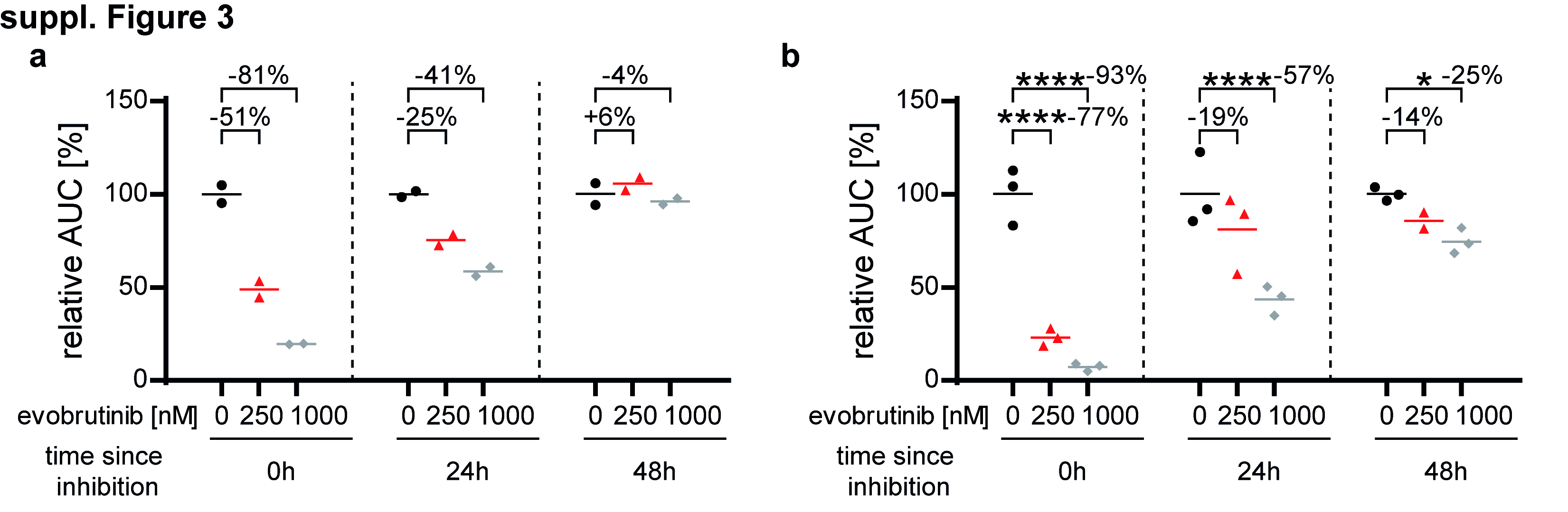

Supplement: Supplementary file 4 — Supplementary material 4 (TIFF 14576 kb) [file 401_2020_2204_MOESM4_ESM.tif]

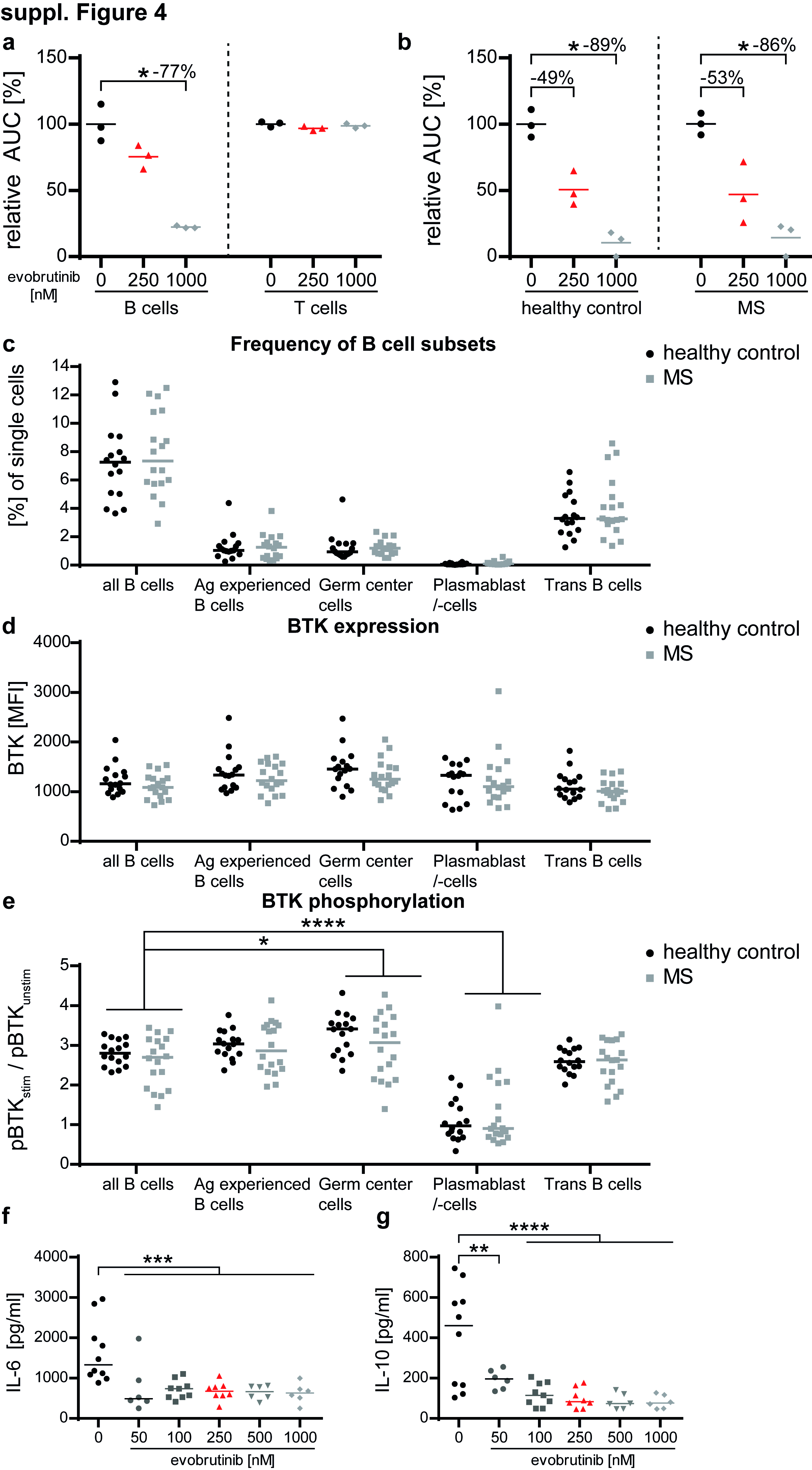

Supplement: Supplementary file 5 — Supplementary material 5 (TIFF 41591 kb) [file 401_2020_2204_MOESM5_ESM.tif]
